# Editorial: Biotechnology and Bioengineering Applications for Egg-Derived Biomaterials

**DOI:** 10.3389/fbioe.2021.756058

**Published:** 2021-09-20

**Authors:** Tamer A. E. Ahmed, Chibuike C. Udenigwe, Ahmed Gomaa

**Affiliations:** ^1^ Department of Cellular and Molecular Medicine, Faculty of Medicine, University of Ottawa, Ottawa, ON, Canada; ^2^ School of Nutrition Sciences, Faculty of Health Sciences, University of Ottawa, Ottawa, ON, Canada; ^3^ Nutrition and Food Science Department, National Research Centre, Cairo, Egypt

**Keywords:** editorial, biotechnology, bioengineering, egg, biomaterial, eggshell, membrane, applications

The world generates around 71 million metric tons of table eggs annually (Ahmed et al., 2019). About 30% of shell eggs are directed to breaker processing plants that create high quantities of eggshell (ES) and eggshell membrane (ESM) waste (Kulshreshtha et al., 2020). The ES is constituted mainly of CaCO_3_ arranged as a calcite crystal (Stapane et al., 2020), while ESM is a naturally occurring proteinaceous meshwork (Ahmed et al., 2017). The ESM holds the internal egg content and plays a crucial role in the assembly of ES by providing the structural support upon which biomineralization takes place (Ahmed et al., 2019). In combination with ESM, ES contains around 1,000 proteins with diverse functionalities and is considered a promising biomaterial (Ahmed et al., 2017). Numerous studies have been conducted to exploit ES waste for value-added applications (Kulshreshtha et al., 2020). The unique characteristics and abundance of ES and ESM make them promising biomaterials for various biotechnological applications (Cordeiro and Hincke, 2011; Ahmed et al., 2019). The objective of this research topic “Biotechnology and Bioengineering Applications for Egg-Derived Biomaterials” within the scope of the section “Biomaterials” featured by the open-access journal “Frontiers in Bioengineering and Biotechnology” was to provide insight into the new technologies exploring the under-valued ES and the associated ESM. The research topic has a relatively broad theme to highlight the different applications of ES and ESM.

The first three articles featured in this research topic are reviews. The first review article (Baláž et al.) provided an overview of the applications of ES waste in the last 5 years in material sciences. Professor Matej Baláž's (Slovakia) research team described the utilization of ES in different environmentally beneficial processes, including ES-based catalysis in organic synthesis, photocatalysis, conventional heterogeneous catalysis, gasification process, and catalytic oxidation. The review article also covered the applications of ES in electrochemistry (separators, electrodes, and sensors) and therapeutics (both ES and ESM). The utilization of various mechanochemical tools enables nanoscale material to be produced for bioceramic synthesis, building industry (cement production), dehalogenation processes, dental materials, lithium-ion batteries, and preparation of hydrophobic filters, and wastewater treatment.

The second (Ahmed et al.) and third (Ahmed et al.) reviews shed light on the intellectual landscape of biotechnological applications of ES between 2015 and 2020. A survey of the most recent patents in engineering technology for ES screening, separation, and processing was underlined by Ahmed et al. Screening technologies summarized patented inventions that have been developed to monitor ES quality. Washing, sterilization, and separation of ES from liquid egg contents represent the primary processing approaches to generate high-quality ES.

A snapshot of the most recent patents, emphasizing different biotechnological applications of ES waste, including chemical, biomedical, environmental, and engineering technologies, was discussed by Ahmed et al. and is considered a complementary article to Ahmed et al. Developing value-added applications for ES waste is a sustainable waste management approach, as it reduces the consumption of natural resources and recycles a valuable natural biomaterial while minimizing waste production. In addition, such a strategy has an economic impact by reducing the costs associated with the disposal of ES waste.

The last article is original research (Ramasamy et al.) addressing the utilization of alternative biomaterial membrane (human amniotic membrane) to develop nanoparticle-based dressing for wound healing. The human amniotic membrane-based dressing combines some important advantages, such as its anti-inflammatory and antimicrobial properties. In addition, it is non-immunogenic and reduces pain and scar formation. Furthermore, the membrane-based wound dressings are resilient, readily available, and cost-effective with 3–5 years of shelf life. Moreover, nanoparticles derived from the membrane exhibited a broad-spectrum antimicrobial activity and reduced biofilm formation in Gram-negative and Gram-positive bacteria. The researchers optimized the membrane processing to have the potential of natural healing properties.

The dramatic increase in novel screening, separation, collection, and processing technologies reflects the industrial interest to diversify the applications of the low-value ES waste material, and is an essential step in developing advanced technologies that explore new biomedical, chemical, engineering, and environmental applications for ES and the associated membrane. Although the number of ES-derived patents has increased exponentially in the last two decades, continued exploration of innovative top-down strategies is key to amplifying the value of ES. We anticipate that these insights and perspectives will raise awareness in academia, government, and private sector to explore more sustainable approaches and implement solutions that target the poultry industry ES byproduct.
